# Surface-Modified Sewage Sludge-Derived Carbonaceous Catalyst as a Persulfate Activator for Phenol Degradation

**DOI:** 10.3390/ijerph17093286

**Published:** 2020-05-08

**Authors:** Meiling Han, Jin Zhang, Wen Chu, Gongfu Zhou, Jiahao Chen

**Affiliations:** 1 College of Navigation, Dalian Maritime University, Dalian 116026, China; zhougongfudlmu@163.com (G.Z.); chenjiahao_0202@163.com (J.C.); 2 College of Environmental Sciences and Engineering, Dalian Maritime University, Dalian 116026, China; zhangjin@dlmu.edu.cn; 3 ACRE Coking & Engineering Consulting Corporation, MCC, Dalian 116085, China; chuwen4881@hotmail.com

**Keywords:** sewage sludge, carbonaceous, persulfate, phenol, degradation

## Abstract

In this study, a catalytic persulfate oxidation process comprising sodium persulfate (PS) and modified sewage sludge-derived carbonaceous catalysts was tested for the degradation of phenol. Sludge-based biochar was modified by high-temperature treatment combined with hydrochloric acid oxidation. The surface properties of carbonaceous catalysts before and after modification were characterized by elemental analysis, N_2_ isothermal adsorption-desorption, scanning electron microscopy (SEM) and Fourier transform infrared (FTIR) spectroscopy. The effects of reaction parameters including catalyst dosage, PS/phenol molar ratio, initial pH and reaction temperature on the degradation rate of phenol were investigated. The kinetics of phenol transformation was explored and the reaction rate appeared pseudo first-order kinetics. In SS-600-HCl/PS system, 91% phenol could be efficiently degraded under certain reaction conditions ([phenol]_0_ = 100 mg/L, catalyst dosage = 0.8 g/L, PS/phenol molar ratio = 3/1, pH = 7, 25 °C) in 180 min. Thus, the results showed that the modified sewage sludge-derived carbonaceous catalyst had a better ability to activate PS for phenol degradation.

## 1. Introduction

Phenol and its derivatives, considered as among the most toxic pollutants from industrial wastewater, are widely used in the coal chemical industry, oil refining, petrochemical, papermaking and the pharmaceutical industry [1,2,3]. The United States Environmental Protection Agency (USEPA) has listed phenol as a priority pollutant. Meanwhile, the World Health Organization (WHO) recommends that the allowable concentration of phenol in drinking water is 1 µg/L. Because of its great harm to human beings and difficulty to degrade, the research on the treatment of this kind of organic wastewater has always been a hot topic in the field of industrial water treatment [4,5,6].

Advanced oxidation processes (AOPs) are one of the effective methods to treat refractory organic wastewater. In recent years, various AOP technologies have been widely applied in wastewater treatment, such as catalytic ozonation [7], catalytic wet oxidation [8], electric catalytic oxidation [9], catalytic hydrogen peroxide oxidation [10], catalytic persulfate oxidation [11], and so on. Among them, as an alternative to hydroxyl radicals, sulfate radical contains a lone pair of electrons on the outside, which has a long half-life, strong oxidation property and wide operative pH range [12]. As it has more advantages in practical industrial wastewater treatment, the sulfate radical was often used to degrade organic pollutants [13]. However, persulfate has low oxidation capacity. Sulfate radical with higher reduction potential (SO_4_^−^, E^0^ = 2.60 V) should be obtained through activation by heating [14], ultraviolet (UV) radiation [15], from transition metals [16] and other methods, which either requires high energy input, harsh reaction conditions or produces metal toxicity. Therefore, developing efficient, economical and non-toxic catalysts as persulfate activators is an environmentally friendly way for AOPs to treat contaminants.

Recently, carbonaceous materials have attracted a lot of attention for persulfate activation because of their advantages of low cost, eco-friendly, high specific surface area, and abundant functional groups on the surface. It has been widely investigated that metal-free carbon materials including activated carbon [17,18], carbon nanotubes [19], mesoporous carbon [20,21], graphene [22] and biochar [23,24] can effectively catalyze persulfate to produce free radicals for the degradation of contaminants. Meanwhile, with the increase of the output of residual sludge in sewage treatment plants, investigating how to use sludge-derived biochars as persulfate activators would be an excellent choice [25]. The functional groups on the carbon surface, such as carboxyl, carbonyl, lactones, phenols and quinones, can significantly influence the catalytic properties [26]. Various modification methods have been reported to control the potential of functional groups, including pyrolysis treatment [27], alkali treatment [28], acid treatment [29] and nitrogenation treatment [30].

However, to the best of our knowledge, only a few studies have been carried out for the catalytic persulfate oxidation reaction with modified sludge-derived biochar to degrade phenol [31]. Therefore, the aim of this study is to demonstrate the potential of sewage sludge-derived carbonaceous catalysts (SCs) as persulfate activators for phenol degradation. In this paper, the sludge based biochar was modified by high temperature treatment combined with hydrochloric acid oxidation, and the physical and chemical properties of carbonaceous materials before and after the modification were characterized. At the same time, the modified catalysts were used to activate PS to degrade phenol. The catalytic activation performance of sludge-based carbonaceous catalysts under different conditions was studied. The effects of reaction parameters including catalyst dosage, PS concentration, initial pH value and temperature were examined. The kinetics of phenol transformation were explored and the reaction rate appeared to show pseudo first-order kinetics. Furthermore, the stability of the catalyst was evaluated by three recycling experiments with the used and regenerated catalysts.

## 2. Materials and Methods

### 2.1. Materials and Reagents

The sewage sludge was collected from a wastewater treatment plant (WWTP) in Dalian (China). Phenol, sodium persulfate (PS), HCl, NaOH, H_2_SO_4_, Ethanol and other chemicals were purchased from Damao Chemical Reagent Co., Ltd. (Tianjin, China). All the chemicals were of reagent grade and used without further purification. High-purity water with a resistivity of 18.2 mΩ cm was used to prepare the solutions.

### 2.2. Preparation and Characterization of the Sludge-Derived Carbonaceous Catalysts (SCs)

The sewage sludge was dried at 105 °C to constant weight and denoted as SS. Then, SS was carbonized at 600 °C for 4 h. The pyrolysis process was carried out in a tubular furnace (heating rate: 5 °C min^−1^) in a N_2_ atmosphere to reach the activation temperature. After the furnace had cooled to room temperature, the SS-600 was obtained. Then, the samples were impregnated with the same volume hydrochloric acid (20.5 wt.%) for 24 h at room temperature, and thoroughly washed with deionized water until the pH reached 6–7 to obtain SS-600-HCl. Finally, the SS-600-HCl was dried at 105 °C for 24 h.

The element composition was analyzed by an elemental analyzer (Vario EL cube, Elementar, Germany). The ash content (weight ratio, wt.%) was measured according to ASTM D 3176 [32]. The specific surface areas of SC was measured by Brunauer–Emmett–Teller (BET) methods and was determined with N_2_ adsorption-desorption isotherms at 77 K using a surface area analyzer instrument (Micromeritics ASAP 2020, Norcross, GA, USA). The sample was degassed for 3 h at 573 K before measurement. The total pore volume (*V_p_*) was obtained from the N_2_ amount adsorbed at a relative pressure close to unity. The pore size (*L*_0_) distribution was determined by the Barrett-Joyner-Halenda (BJH) method. Scanning electron microscopy (SEM) experiments were performed on a scanning electron microscope (Hitachi SU8200, Tokyo, Japan). Functional groups on the SCs were analyzed by Fourier Transform Infrared Spectroscopy (FTIR) (Bruker ALPHA, Ettlingen, Germany).

### 2.3. Batch Experiments and Analytical Methods

The experiment was carried out in a 250 mL glass conical beaker with phenol (150 mL, 100 mg/L). The beaker was stirred by a magnetic stirrer (500 rpm) at 25 °C. The experiment was divided into two parts: adsorption experiment and degradation experiment. In the adsorption experiment, the catalyst dosage was 0.8 g/L. The solution was sampled every 10 min and measured concentration after filtration process with 0.45 μm membrane. In the degradation experiment, 114 mg of PS was added into the solution and the influence of catalysts was determined. The molar ratio of PS/phenol was 3/1, the initial solution pH was 7.0 and the solution pH was not controlled during the degradation study. The sample was filtered by a 0.45 μm filter and quenched with ethanol for analysis. Then the optimal conditions of catalyst dosage, molar ratio of PS/phenol, initial pH value and temperature were investigated. The initial solution pH was adjusted with 1 M NaOH or 1 M H_2_SO_4_. Phenol concentration was analyzed by an ultraviolet–visible (UV–vis) spectrophotometer (UV-1900PC, AOE Instruments, Shanghai, China) using a wavelength of 270 nm. The phenol conversion was calculated by the following formula (Equation (1)):

Phenol conversion (%) = (C_0_ −C_t_)/C_0_ × 100 (1)

where C_0_ is the initial absorbance of phenol solution, C_t_ is the absorbance of phenol solution at the reaction time (t).

## 3. Results and Discussion

### 3.1. Characterization of SCs

#### 3.1.1. Composition Analysis

Table 1 presents the composition analysis of sewage sludge, pyrolysis-treated sludge, and HCl-treated sludge. The elements analysis included C, H, N and S. It is obvious that SS-600-HCl had the highest C percentage composition, with 30.07 wt.%, but the lowest ash content, with 9.40 wt.%. The results indicated that SS-600-HCl could have more potential active sites and porous structures, which could promote the efficient oxidation reaction.

#### 3.1.2. Nitrogen Adsorption-Desorption Isotherms and Pore Size Distributions

**Figure 1:**
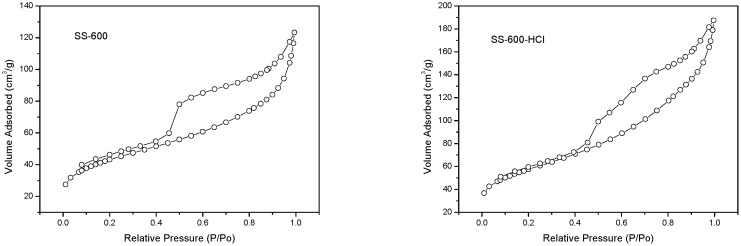
N_2_ adsorption-desorption isotherms of SS-600 and SS-600-HCl.

**Figure 2:**
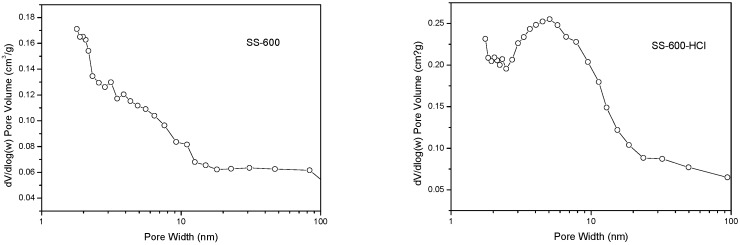
Pore size distributions of SS-600 and SS-600-HCl.

The specific surface area, total pore volume and average pore size of SCs were shown in Table 2. A higher specific surface area (197 vs. 147 m^2^/g) and a larger pore volume (0.29 vs. 0.19 cm^3^/g) of SS-600-HCl than SS-600 were observed. This implied that HCl activation had resulted in more abundant specific surface area and pore volume on the catalyst. Average pore size (5.89 vs. 5.21 nm) of SS-600-HCl and SS-600 indicated the mesoporous texture of catalysts. The isothermal adsorption/desorption curves of SCs were shown in Figure 1. A IV type isotherm with a H4 type hysteresis loop was exhibited, which implied a mesoporous structure, corresponding with average pore size [33]. The pore size distribution of SCs were shown in Figure 2. SS-600-HCl had some larger pores than SS-600, centered at 5 nm, which indicated that the HCl activation could increase the pore of catalyst, even a more concentrated pore size distribution.

#### 3.1.3. Scanning Electron Microscopy (SEM) Analysis

**Figure 3:**
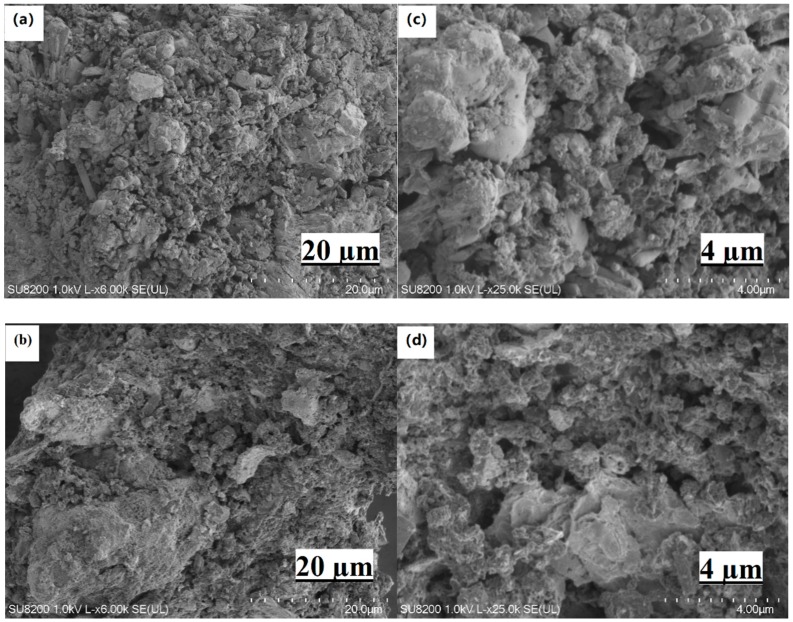
Scanning electron microscopy (SEM) images of SS-600 (**a**,**c**) and SS-600-HCl (**b**,**d**).

The morphologies and microstructures of SCs before and after HCl treatment were further studied by SEM. The SEM images of SS-600 and SS-600-HCl were shown in Figure 3. It can be seen that the SCs surface had a roughness, slit-shape type structure and a large number of pores. Compared with SS-600, SS-600-HCl had more pores and larger pore size on the surface, and structural defects and faults on the surface were relatively obvious, which was consistent with the specific surface area data in Table 2. The above results might be caused by acid oxidation treatment of oxygen-containing groups on the surface and at the orifice of carbonaceous material. Therefore, the surface characteristics of SS-600 was improved by the HCl treatment. After HCl treatment, more uniform mesoporous pores were formed on the surface of SS-600-HCl.

#### 3.1.4. Fourier Transform Infrared (FTIR) Spectroscopy Analysis

**Figure 4:**
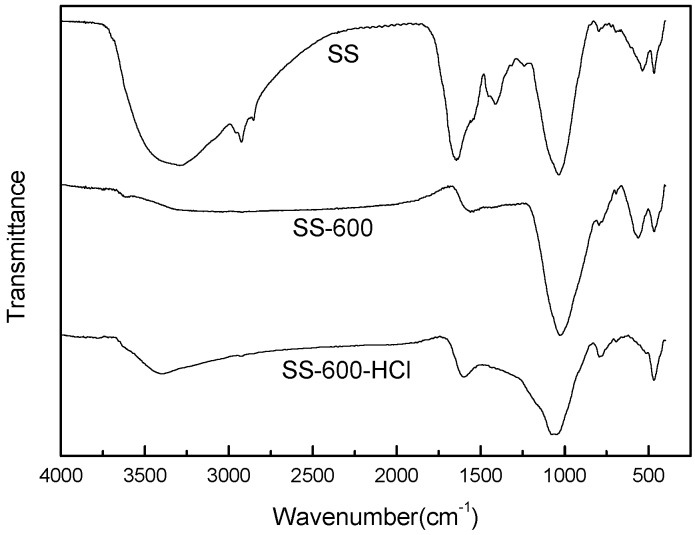
Fourier transform infrared (FTIR) spectra of raw sludge and SCs.

With the change of pyrolysis and acid treatment condition, the surface groups of biochar changed, and the FTIR spectra of raw sludge and SCs were shown in Figure 4. The prominent broad peak at 3300–3500 cm^−1^ was attributed to the presence of -OH groups [34]. The peak of SCs treated by pyrolysis and acid was obviously weaker than that of raw sludge. The vibration peaks of 2926 cm^−1^ and 1412 cm^−1^ are -CH_2_- and -CH_3_ groups [35]. With the progress of pyrolysis, organic substances such as cellulose in the sludge decomposed, resulting in the reduction of the absorption peak strength of alkyl groups. The bands observed in the 1600–1650 cm^−1^ region belong to aromatic C=C and C=O functional groups [36], whose strength decreased with the progress of pyrolysis. The strong adsorption peak at 1020–1050 cm^−1^ was related to the stretching vibration of the C–O bond [35], which showed high strength in the spectra of all carbonaceous materials [37].

### 3.2. Performance of SCs on Phenol Degradation

**Figure 5:**
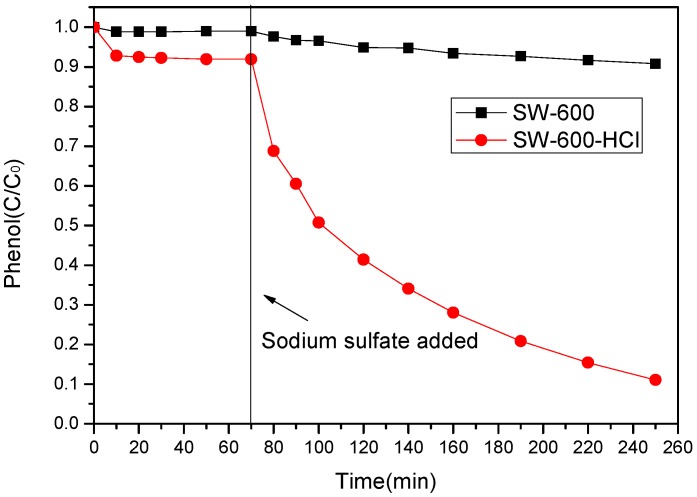
Degradation of phenol in batch reactor through adsorption and catalytic oxidation by SCs/sodium persulfate (PS) system ([phenol]_0_ = 100 mg/L, catalysts dosage = 0.8 g/L, PS/phenol molar ratio = 3/1, pH = 7, 25 °C).

The adsorption and degradation performance of phenol in a SCs/PS system were carried out and results are shown in Figure 5. Without adding PS, the adsorption removal of phenol by SS-600 and SS-600-HCl was 1% and 8%, respectively, in 70 min. A slight enhancement in the adsorption of phenol by SS-600-HCl might be due to its larger surface area. However, it is apparent that the adsorption ability of the two catalysts was weak, while a small amount of phenol was adsorbed by both (less than 10%). So the effect of adsorption on the experiment could be ignored.

From Figure 5, the catalytic oxidation performance of the SCs/PS system was observed. When the initial concentration of phenol was 100 mg/L and the molar ratio of PS/phenol was 3/1, a degradation ratio of phenol of 89% could be achieved with the SS-600-HCl/PS system while there was only 9% phenol removal with the SS-600/PS system in 180 min. By contrast, SS-600-HCl showed an extremely higher catalytic activity. The experimental results indicated that, according to Table 2, the higher specific surface area would increase the area of contact with the oxidation in the catalytic reaction [38]. Furthermore, the difference in conversion is significantly higher than the difference in surface area. This suggests that the specific surface sites on the treated material are more active than those on the untreated sample. A previous study had pointed out that the catalyst with a large surface area could provide more active sites and produce more radicals for catalytic oxidation of phenol [39]. Thus, the activity of the carbonaceous catalyst modified by hydrochloric acid oxidation was significantly higher than that of carbonaceous catalyst treated by high-temperature carbonization alone.

### 3.3. Effect of Reaction Parameters on Phenol Degradation

#### 3.3.1. Effect of SS-600-HCl Dosage

**Figure 6:**
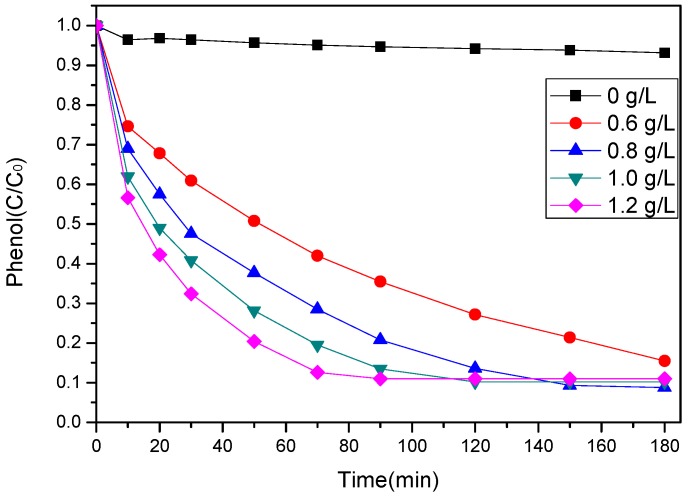
Effect of SS-600-HCl dosage on phenol degradation ([phenol]_0_ = 100 mg/L, PS/phenol molar ratio = 3/1, pH = 7, 25 °C).

A comparison of phenol degradation in the SS-600-HCl/PS system with different catalyst dosages (0, 0.6, 0.8, 1 and 1.2 g/L) is shown in Figure 6. The appropriate amount of phenol (100 mg/L) and PS (PS/phenol molar ratio = 3/1) was fixed. As can be seen, only 7% of phenol was removed by PS in the absence of any catalyst after 180 min. This result proved that PS itself could not produce sulfate radicals to induce significant oxidation of phenol [40]; 84% of phenol removal efficiency was achieved in the presence of a 0.6 g/L catalyst. The results showed that the addition of the catalyst could significantly activate PS and showed good catalytic activation ability. With the increase of catalyst dosage, the degradation rate of phenol increased rapidly. When the catalyst dosage was increased to 0.8 g/L, the conversion of phenol reached 91%. However, by further increasing the dosage of the catalyst, the degradation efficiency of phenol was not improved obviously. The results indicated that the presence of catalyst was beneficial to sulfate radical formation and improved the degradation performance of phenol. In addition, higher adsorption capacity and more active sites for PS activation can be provided as the catalyst dosage is increased.

#### 3.3.2. Effect of Sodium Persulfate (PS) Dosage

**Figure 7:**
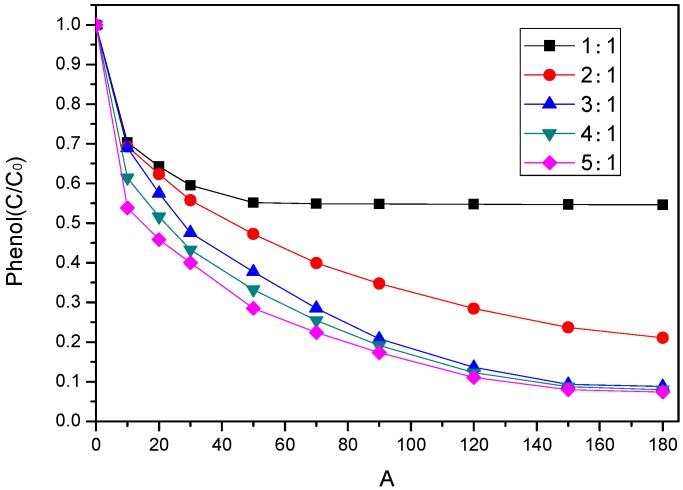
Effect of PS dosage on phenol degradation ([phenol]_0_ = 100 mg/L, catalyst dosage = 0.8 g/L, pH = 7, 25 °C).

The effect of different molar ratios of PS/phenol (1/1, 2/1, 3/1, 4/1, and 5/1) on phenol degradation is evaluated in Figure 7. The experimental results showed that the phenol degradation improved from 45% to 93% when the PS/phenol molar ratio increased from 1/1 to 5/1. It indicated that higher PS concentration could increase the removal efficiency of phenol. It was inferred that the higher PS concentration is, the more free radicals could be generated; 91% phenol removal appeared at 180 min after increasing the amount of molar ratio of PS/phenol to 3/1. However, by further increasing PS concentration, phenol degradation efficiency appeared to be slightly enhanced. The reason might be that sulfate radical produced after activation reacted with excessive PS, which further inhibits the reaction [41].

#### 3.3.3. Effect of Initial pH

**Figure 8:**
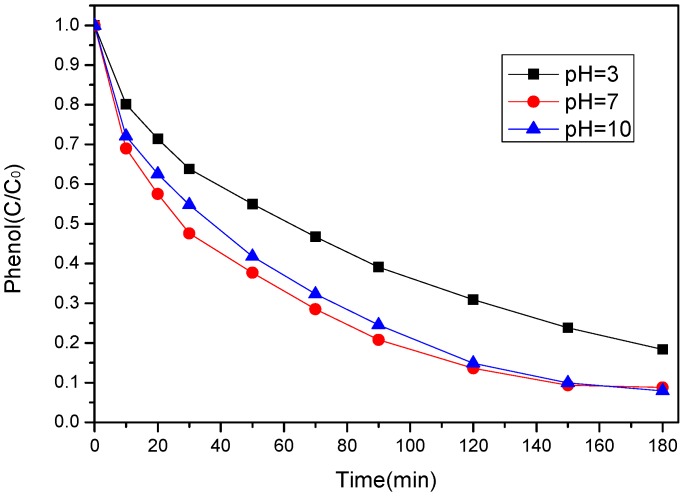
Effect of initial pH on phenol degradation ([phenol]_0_ = 100 mg/L, catalyst dosage = 0.8 g/L, PS/phenol molar ratio = 3/1, 25 °C).

The influence of initial pH was investigated at three values, i.e., 3, 7 and 10. As shown in Figure 8, high conversions and removal rates of phenol were maintained at pH 7 and pH = 10. Then we found out that the pH value of phenol solutions were decreased from 7.0 to 4.0 and 10.0 to 7.1 after the PS was added, which is consistent with the previous study [42]. Therefore, the reactions were under the pH of 4.0 and 7.1. It has been reported that sulfate radicals can be motivated easily under a wide range of pH level (3–9), while OH^−^/H_2_O is oxidized into OH by SO_4_^−^ at a moderate rate. So a relatively high degradation efficiency was maintained by the presence of both SO4^−^ (dominator) and OH [43]. However, with the addition of PS, the pH value was decreased from 3.0 to 1.0. It can be seen that the performance of the SS-600-HCl/PS system on phenol degradation decreased at this pH level. Previous studies pointed out that the process was impeded by inactivation of PS at extreme acidic pH [44]. Thus, the initial acidic condition will be of no benefit to the system. These results indicated that normal initial pH levels would be adaptable to the SS-600-HCl/PS system.

#### 3.3.4. Effect of Temperature

**Figure 9:**
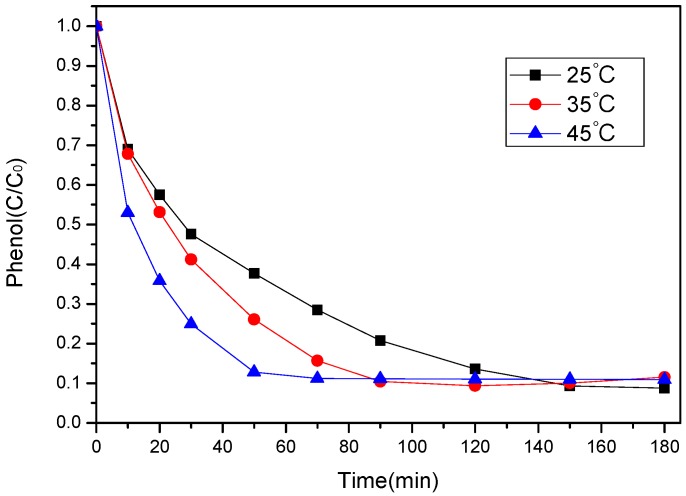
Effect of temperature on phenol degradation ([phenol]_0_ = 100 mg/L, catalyst dosage = 0.8 g/L, PS/phenol molar ratio = 3/1, pH = 7).

The influence of various temperatures (25, 35, and 45 °C) on phenol degradation is shown in Figure 9. As can be seen, phenol conversion rate could reach about 90% at all reaction temperatures within 180 min. Furthermore, phenol removal was fast and achieved 87% at temperature of 45 °C in 50 min but no further significant increase with reaction time prolonging. At the same duration, at temperature of 25 °C and 35 °C, phenol removal only achieved 62% and 74%, respectively.

**Figure 10:**
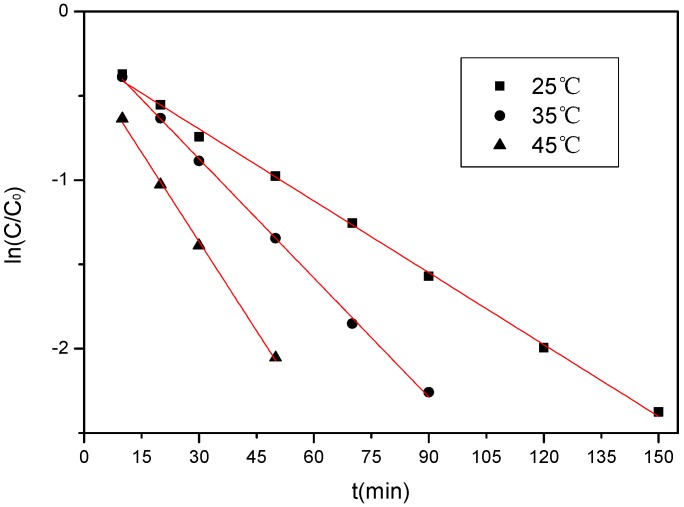
Pseudo-first-order kinetic model of phenol degradation at 25, 35, and 45 °C.

Although 90% phenol degradation could be achieved within 180 min regardless of the reaction temperature, the initial rate increased with increasing temperature. Thus, the kinetics of phenol transformation were explored. A general pseudo first-order kinetic model for phenol degradation was employed, as shown in the equation below:

ln (C/C_0_) = −k⋅t (2)

where k is the apparent first order rate constant of phenol removal, C is the concentration of phenol at various time (t). C_0_ is the initial phenol concentration. Using this model, ln (C/C_0_) versus time (t) produced straight lines as shown in Figure 10. The reaction rate constants at varying temperatures were shown in Table 3. It can be seen that rate constant would increase as the temperature increased.

### 3.4. Mechanism Analysis of Phenol Degradation by SS-600-HCl/PS

**Figure 11:**
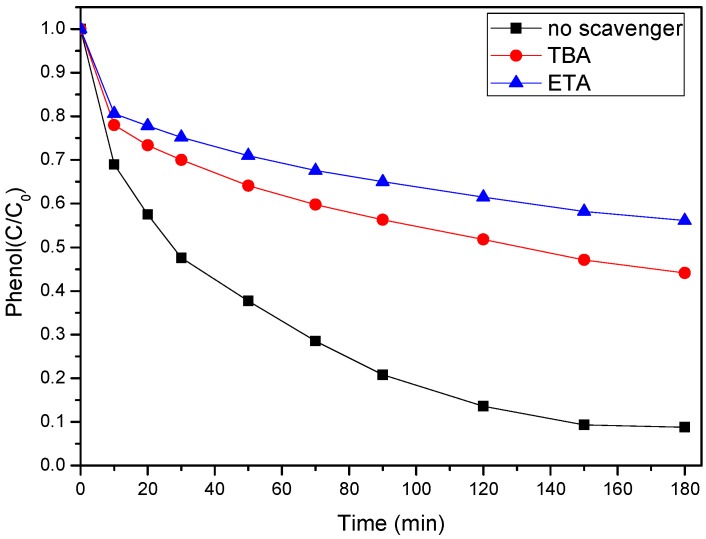
The effect of radical scavengers on phenol degradation ([phenol]_0_ = 100 mg/L, catalyst dosage = 0.8 g/L, PS/phenol molar ratio = 3/1, pH = 7, 25 °C).

To investigate the reaction mechanism of phenol degradation by SS-600-HCl/PS, radical scavenger tests were carried out and the results are shown in Figure 11. It is widely accepted that SO4^−^ and HO are the main oxidative radicals in most PS activation processes. In many radical studies, tert-butyl alcohol (TBA) was reported as the trapping agent of HO, while ethanol (ETA) was used as the trapping agent of SO4^−^ and HO [45]. As it can be noticed that the addition of TBA (3 M) and ETA (3 M) showed strong inhibitory effects on the SS-600-HCl/PS system for phenol degradation. Furthermore, ETA has a stronger inhibitory effect than TBA. The conversion of phenol decreased from 91.2% to 55.9% and 43.9%, respectively. Thus, this confirmed that radical oxidation was the dominant pathway of the SS-600-HCl/PS system for phenol degradation, which was also demonstrated in the precious study on PS radical activation processes [46]. Above all, it was demonstrated that SS-600-HCl could efficiently activate PS for phenol degradation through radical pathways dominated by SO4^−^ and HO.

### 3.5. Stability and Reuse of SS-600-HCl Catalyst

**Figure 12:**
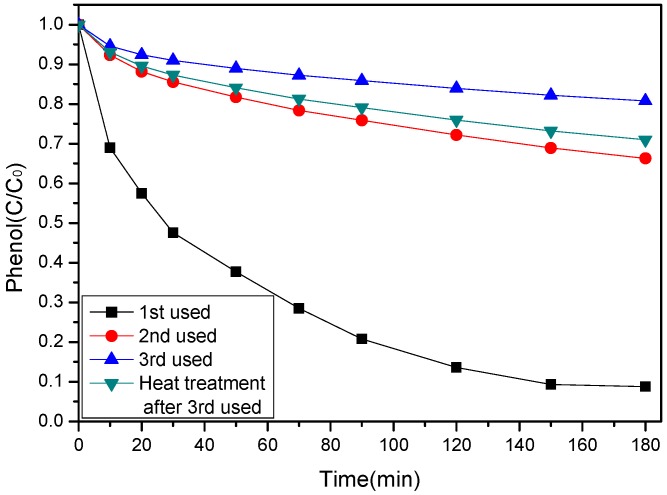
Reusability of SS-600-HCl catalyst on phenol degradation ([phenol]_0_ = 100 mg/L, catalyst dosage = 0.8 g/L, PS/phenol molar ratio = 3/1, pH = 7, 25 °C).

The stability of a catalyst is always crucial for its practical application. Therefore, the reuse performance of SS-600-HCl catalyst was evaluated on a SS-600-HCl/PS system. After each reaction, the catalyst was filtered and washed three times and dried at 105 °C for 12 h. As can be seen from Figure 12, the SS-600-HCl lost catalytic activity significantly after repeated used. After 2 times and 3 times of reuse, the removal rates of phenol were nearly 35% and 20% for 180 min in a SS-600-HCl/PS system, while that of fresh SS-600-HCl in a SS-600-HCl/PS system was 91%. It can be seen that SS-600-HCl was almost completely deactivated after the third time it was used. Then the deactivated SS-600-HCl was treated at 500 °C in N_2_ for 1 h, and its activity was recovered. However, only about 30% phenol could be removed by this catalyst after 180 min reaction in SS-600-HCl/PS system. Therefore, the stability of the present sludge-derived biochar catalyst was not comparable to that of reported metal-based catalysts [47].

**Figure 13:**
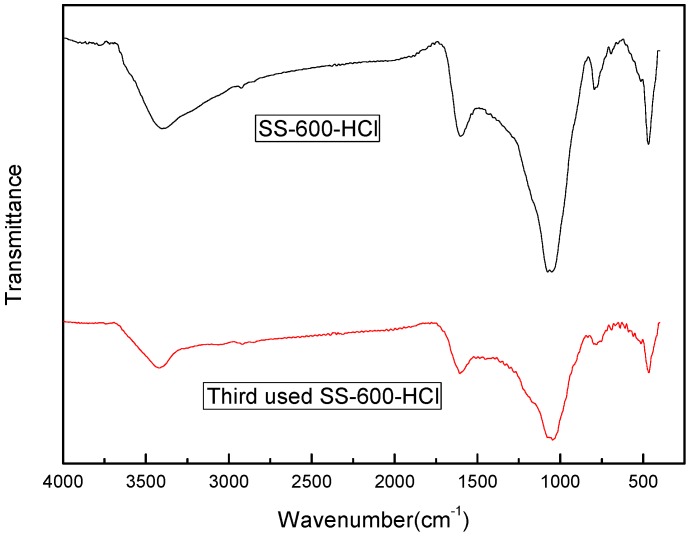
FTIR spectra of the fresh and the SS-600-HCl used three times.

In order to analyze the deactivation mechanism of SS-600-HCl in the reaction, the specific surface area and pore structure of the fresh catalyst, the catalyst used three times, and the heat-treated regenerated catalyst were tested. The results are shown in Table 4. The specific surface area of SS-600-HCl decreased from 197 m^2^/g before use to 3 m^2^/g. The total pore volume decreased significantly, but the average pore size decreased slightly. FTIR spectra of the fresh and the three-times used SS-600-HCl are shown in Figure 13. The decrease of peak strength indicated that active functional groups on the surface of catalyst decreased. Thus, it can be inferred from the above results, after catalytic oxidation reaction, phenol and its degradation products might be adsorbed on the surface of the catalyst, decreasing the specific surface area and covering a large number of active sites. A large number of molecules entered pores, which significantly reduced the pore area and volume. However, after regeneration by heat treatment, the specific surface area, the total pore volume and the average pore size of SS-600-HCl increased from 3 m^2^/g, 0.005 cm^3^/g and 5.71 nm to 9 m^2^/g, 0.043 cm^3^/g and 19.09 nm. This means that the activity of catalyst was partially recovered by heat treatment. The results showed that only a few adsorbed substances on the surface of the catalyst desorbed, which slightly increased the specific surface area of the catalyst and restored part of active sites on the surface. Therefore, the inactivation of SS-600-HCl might be caused by its pore structural change and surface being slightly covered by reactant molecules and product molecules.

## 4. Conclusions

This study comprehensively investigated the reactivity of an SCs/PS system on phenol degradation. With the help of composition analysis, N_2_ adsorption/desorption, SEM and FTIR, the surface properties and the catalytic ability of SCs through pyrolysis treatment and hydrochloric acid modification were evaluated. The performance test of SCs identified the relationship between the catalytic ability and modification methods. Acid modification had a promoting effect on the catalytic ability of the catalyst in the SCs/PS system. It was also found that phenol removal efficiency was influenced by catalyst dosage, PS concentration, initial pH value and temperature. The phenol transformed reaction rate appeared to show pseudo first-order kinetics. In SS-600-HCl/PS system, 91% phenol could be efficiently degraded under certain reaction conditions ([phenol]_0_ = 100 mg/L, catalyst dosage = 0.8 g/L, PS/phenol molar ratio = 3/1, pH = 7, 25 °C) in 180 min. Furthermore, the stability of the catalyst was measured and results showed that adsorption of intermediates and structural change resulted in deactivation of the catalyst and regeneration by calcination could partially recover the catalytic activity.

## Figures and Tables

**Table 1:** Elemental analysis and ash content of sludge-derived carbonaceous catalysts (SCs).

Sample	C (%)	H (%)	N (%)	S (%)	Ash (%)
SS	24.71	4.01	4.15	0.354	53.00
SS-600	16.97	0.522	1.54	0.679	13.60
SS-600-HCl	30.07	0.904	2.97	1.23	9.40

**Table 2:** Structural properties of SCs.

Sample	S_BET_ (m^2^/g)	Total Pore Volume (*V_p_*) (cm^3^/g)	Pore Size (*L*_0_) (nm)
SS-600	147	0.19	5.21
SS-600-HCl	197	0.29	5.89

**Table 3:** Pseudo-first-order kinetic parameters of phenol degradation at 25, 35, and 45 °C.

t (°C)	k_obs_ (min^−^^1^)	R^2^
25	0.01421	0.99819
35	0.02358	0.99884
45	0.03527	0.99776

**Table 4:** Textural characteristics of SCs produced from sewage sludge.

Sample	*S*_BET_ (m^2^/g)	*V_t_* (cm^3^/g)	*L*_0_ (nm)
Fresh SS-600-HCl	197	0.29	5.89
Three times used SS-600-HCl	3	0.005	5.71
Regenerated SS-600-HCl	9	0.043	19.09
